# Variables associated with the relationship between obesity and mental health among university students in the Gulf Cooperation Council countries: a systematic review

**DOI:** 10.3389/fpubh.2024.1411229

**Published:** 2024-08-29

**Authors:** Amjad Joma, Samer Abuzerr, Sharif Alsoudi

**Affiliations:** ^1^Associate Professor in Educational Psychology, Faculty of Art and Humanities, A’Sharqiyah University, Ibra, Oman; ^2^Department of Medical Sciences, University College of Science and Technology, Gaza Strip, Palestine; ^3^Assistance Professor in Mesurerment and Evaluation, Faculty of Art and Humanities, A’Sharqiyah University, Ibra, Oman

**Keywords:** obesity, mental health, university students, Gulf Cooperation Council countries, systematic review

## Abstract

**Background:**

Various factors, including dietary habits, lifestyle choices, socio-economic status, cultural attitudes, academic stress, and access to mental health support services, influence the relationship between obesity and mental health among university students in Gulf Cooperation Council (GCC) countries. Understanding these complex interactions is crucial for developing effective interventions to promote both physical and mental well-being among students in the region. Therefore, this systematic review aims at understanding the variables associated with the relationship between obesity and mental health among university students in the (GCC) countries.

**Methods:**

This systematic review protocol was registered with the International Prospective Register of Systematic Reviews (PROSPERO) [CRD42024517806]. We conducted a systematic literature search using electronic databases including PubMed, PsycINFO, Scopus, CINAHL, Web of Science, and Google Scholar to identify relevant studies published up to 28-February-2024. A narrative synthesis approach was employed to summarize the findings of included studies. Data were synthesized according to predefined themes related to variables associated with the relationship between obesity and mental health among university students in GCC countries.

**Results:**

The prevalence of overweight and obesity among university students in GCC countries is alarmingly high, with a mean rate of 29.4%. Depression, anxiety, and body image dissatisfaction are significantly associated with obesity in this population. Poor sleep patterns are both a predictor and a consequence of obesity-related mental health issues. Socio-cultural factors play a crucial role in shaping students’ perceptions of body image and mental health.

**Conclusion:**

These findings highlight the importance of comprehensive approaches to address the intertwined nature of obesity and mental health in this population, necessitating targeted interventions and further research efforts.

## Introduction

Obesity and mental health disorders are two significant public health concerns worldwide, with their prevalence steadily rising over the past few decades. In recent years, researchers have begun to explore the complex interplay between these two conditions, recognizing the bidirectional relationship that exists. Traditional views of obesity as a physical health issue have given way to emerging evidence that links it closely to various mental health disorders such as depression, anxiety, and eating disorders (1, 2).

The co-occurrence of obesity and mental health problems presents a considerable challenge for healthcare systems globally, highlighting the need for comprehensive interventions that address both physical and psychological well-being.

Despite the growing body of research on the association between obesity and mental health, there remains a paucity of studies focusing on specific populations, such as university students in the Gulf Cooperation Council (GCC) countries. The GCC countries, including Bahrain, Kuwait, Oman, Qatar, Saudi Arabia, and the United Arab Emirates, have experienced rapid economic development and urbanization in recent decades, leading to significant changes in lifestyle and dietary habits (3). These changes have contributed to an increasing prevalence of obesity and related comorbidities within the region (4).

Furthermore, university students represent a unique population subgroup characterized by distinct socio-cultural factors, lifestyle patterns, and stressors that may influence both obesity and mental health outcomes (5, 6). Academic pressures, peer relationships, and lifestyle choices, along with the transition from adolescence to adulthood, can significantly impact students’ physical and psychological well-being (7). Thus, understanding the relationship between obesity and mental health among university students in the GCC countries is crucial for developing targeted interventions and promoting holistic approaches to student health and wellness.

This systematic review aims to comprehensively examine the variables associated with the relationship between obesity and mental health among university students in the GCC countries. By synthesizing existing evidence from empirical studies, we seek to elucidate the various factors that contribute to the co-occurrence of these conditions and identify potential avenues for intervention and prevention. The findings of this review will not only contribute to the existing literature, but also inform healthcare practitioners, policymakers, and educators about the unique challenges faced by university students in the GCC region and the importance of addressing both physical and mental health in this population.

## Methods

### Protocol registration and search strategy

Prior to conducting the review, we registered this systematic review protocol with the International Prospective Register of Systematic Reviews (PROSPERO; CRD42024517806) to enhance transparency and reduce the risk of bias. We conducted a systematic literature search using electronic databases including PubMed, PsycINFO, Scopus, CINAHL, Web of Science, and Google Scholar to identify relevant studies published up to February 28, 2024. The search strategy utilized a combination of keywords related to “obesity,” “mental health,” “university students,” and “Gulf Cooperation Council countries” (Appendix A). Additionally, we hand-searched the reference lists of identified articles and relevant systematic reviews for additional studies.

### Study selection criteria

Eligible studies were selected based on predefined inclusion criteria. We included studies that were conducted among university students in one or more GCC countries (Bahrain, Kuwait, Oman, Qatar, Saudi Arabia, and the United Arab Emirates), studies investigated the relationship between obesity (defined by body mass index or other measures) and mental health outcomes (e.g., depression, anxiety, eating disorders), studies employed observational study designs or intervention studies with a comparison group, and studies published in peer-reviewed journals in English.

### Data extraction

Two independent authors screened the titles and abstracts of all identified studies to assess their eligibility for inclusion. Full-text articles of potentially eligible studies were retrieved and independently assessed for eligibility based on the inclusion criteria. Discrepancies were resolved through discussion or by consulting a third author.

Data extraction was performed independently by two authors using a standardized data extraction form. Extracted data included study characteristics (e.g., author(s), year of publication, country/setting, study design), participant characteristics (e.g., sample size, age, and gender distribution), measures of obesity and mental health outcomes, key findings, future implication or recommendations, and scoring (Tables 1, 2). The screening methods for the articles were managed using EndNote V.X8 software.

**Table 1:** Baseline characteristics of included studies.

First author name	Year of publication	Country/setting	Study design	Sample size	Age (Mean ± Std.) year	Gender distribution (M/F)	Measures of obesity
Al-Sayegh et al. (13)	2020	Kuwait/Kuwait University	Cross-sectional Study	600	20.3 ± 2.6	116/484	BMI = weight [kg]/height [m^2^]
Bener et al. (18)	2006	Qatar/Secondary and high schools	Cross-sectional Study	800	14–19 years	0/800	BMI = weight [kg]/height [m^2^]
Khadri et al. (24)	2020	United Arab Emirates/Emirate of Sharjah	Cross-sectional Study	803	12.8 ± 1.4	406/397	BMI = weight [kg]/height [m^2^]
Dweik et al. (15)	2022	United Arab Emirates/Emirate of Abu Dhabi	Cross-sectional Study	395	14.9 ± 2.07	186/209	BMI = weight [kg]/height [m^2^]
Bener et al. (18)	2006	Qatar/Secondary and high schools	Cross-sectional Study	593	14–19 years	593/0	BMI = weight [kg]/height [m^2^]
Pathath et al. (14)	2017	Saudi Arabia/Different colleges of King Faisal University located in Al-Hasa	Cross-sectional Study	128	18–30 years	60/66	BMI = weight [kg]/height [m^2^]
Alabdullgader et al. (16)	2017	Saudi Arabia/Qassim university, Colleges of Medicine, Dentistry, Pharmacy and Applied Health Sciences	Cross-sectional Study	65	NM	65/0	BMI = weight [kg]/height [m^2^]
Mellal et al. (10)	2014	United Arab Emirates/Abu Dhabi/Al Ain University of Science and Technology	Cross-sectional Study	604	17 -> 31	205/399	BMI = weight [kg]/height [m^2^]
Schulte et al. (11)	2013	United Arab Emirates/United Arab Emirates and Sharjah	Cross-sectional Study	361	19.9 ± 1.90	153/208	BMI = weight [kg]/height [m^2^]

**Table 2:** Summary of key findings on obesity and mental health.

First author name	Year of publication	Measures of mental health	Prevalence of obesity/overweight (%)	Key findings	Quality scoring
Al-Sayegh et al. (13)	2020	Harvard Medical School Special Health Report. Positive Psychology. 2011.	48.1	High prevalence of overweight/obesity among staff and students, suboptimal health indices, lifestyle practices.	High
Bener et al. (18)	2006	Self-Reporting Questionnaire (SRQ-20) for psychopathology	13.4	Extreme dieting linked to body image dissatisfaction, psychological distress.	High
Khadri et al. (24)	2020	Rosenberg Self-esteem Scale	10.0	No significant impact of obesity on self-esteem; variations based on age, brushing frequency.	High
Dweik et al. (15)	2022	Mood Scale Patient Health Questionnaire (MSPHQ)	41.5	Short sleep duration associated with obesity, depressive symptoms.	Medium
Bener et al. (18)	2006	Self-Reporting Questionnaire (SRQ-20) for psychopathology	34.0	Extreme dieting linked to body image dissatisfaction, psychological distress.	High
Pathath et al. (14)	2017	The DASS 21 item self-report questionnaire	26.5	Socio-cultural changes linked to rising obesity rates, need for further research.	Low
Alabdullgader et al. (16)	2017	A self-administered psych-social problems questionnaire	31.2	Mood swings, social pressure, low self-esteem associated with obesity.	Low
Mellal et al. (10)	2014	Self-administered patient’s health questionnaire (PHQ-9)	32.1	Depression linked to age, financial difficulties, perceived weight status.	High
Schulte et al. (11)	2013	The Beck Depression Inventory-II (BDI-II)	27.8	Need for prevention strategies addressing depressive co-morbidity	Medium

### Quality assessment

The methodological quality of included studies was assessed independently by two authors using appropriate tools based on the study design (e.g., Newcastle-Ottawa Scale for cohort and case–control studies, AXIS tool for cross-sectional studies) (8). Studies were evaluated based on criteria such as sample representativeness, measurement validity and reliability, adjustment for potential confounders, and appropriate statistical analysis.

### Data synthesis and analysis

A narrative synthesis approach was employed to summarize the findings of the included studies. The data were synthesized according to predefined themes related to variables associated with the relationship between obesity and mental health among university students in GCC countries.

### Reporting

This systematic review will be reported in accordance with the Preferred Reporting Items for Systematic Reviews and Meta-Analyses (PRISMA) guidelines to ensure transparency and rigor in the reporting of methods and results (Appendix B) (9).

## Results

### Literature search

**Figure 1:**
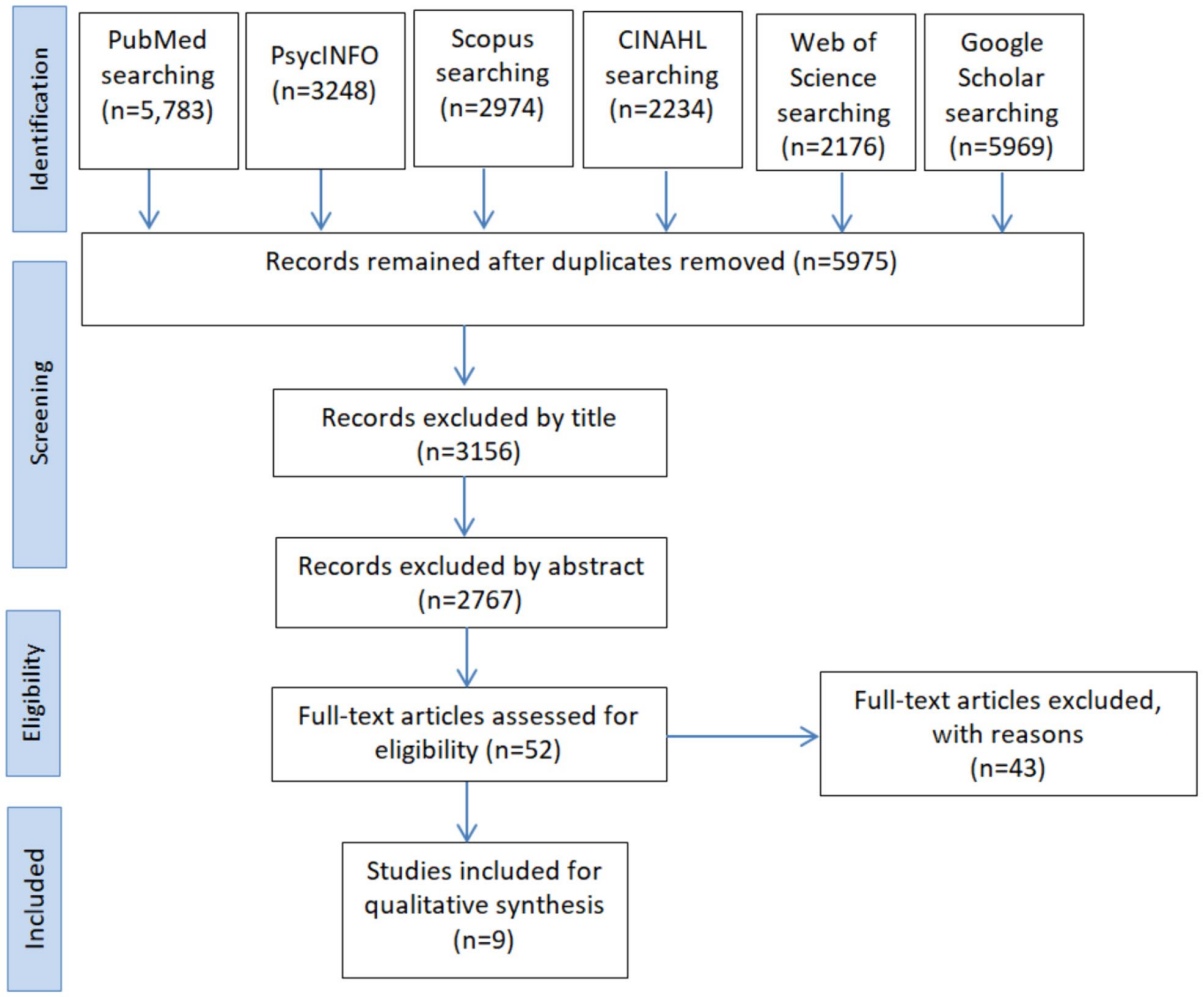
Flowchart detailing the literature search process conducted.

A comprehensive search across multiple databases, including PubMed, PsycINFO, Scopus, CINAHL, Web of Science, and Google Scholar, yielded a total of 24,884 records. After removing duplicates, 5,975 records remained for further evaluation. Title and abstract screening excluded 5,923 records, leaving 52 articles for full-text assessment. Following the eligibility assessment, we excluded 42 articles, which led to the inclusion of 9 studies for qualitative synthesis (Figure 1).

### Characteristics of included studies

The included studies spanned various Gulf Cooperation Council (GCC) countries, including Kuwait, Qatar, the United Arab Emirates (UAE), and Saudi Arabia. These studies employed cross-sectional designs and encompassed sample sizes ranging from 65 to 804 participants. Body mass index (BMI) was the main tool used to measure obesity (Table 1). The Self-Reporting Questionnaire (SRQ-20), mental health was assessed using various tools, including the Self-Reporting Questionnaire (SRQ-20), Rosenberg Self-esteem Scale, Mood Scale Patient Health Questionnaire (MSPHQ), and Beck Depression Inventory-II (BDI-II; Table 2).

### Synthesizing the results

The findings of this systematic review highlight the complex interplay between obesity and mental health among university students in Gulf Cooperation Council (GCC) countries. By synthesizing the results of nine studies, this section aims to elucidate the key variables associated with this relationship, compare the findings with those of other relevant studies, and identify implications for future research and interventions.

### Prevalence of obesity

The mean prevalence rate of overweight and obesity among university students in the included studies was 29.4%. Individual studies reported prevalence rates ranging from 10 to 48.1%.

### Associations between obesity and mental health

#### Depression

Mellal et al. (10) found a significant association between obesity and depression, with obese students reporting higher levels of depressive symptoms as measured by the Patient Health Questionnaire (PHQ-9) (10). Schulte et al. (11) reported that higher BMI was correlated with increased scores on the Beck Depression Inventory-II (BDI-II) (11).

#### Anxiety

Bener et al. (12) indicated that extreme dieting behaviors, prevalent among obese individuals, were associated with heightened anxiety levels as measured by the Self-Reporting Questionnaire (SRQ-20) (12).

#### Body image dissatisfaction

Al-Sayegh et al. (13) and Pathath et al. (14) identified significant correlations between obesity and body image dissatisfaction, with obese students expressing greater dissatisfaction with their body image on the Rosenberg Self-esteem Scale (13, 14).

#### Sleep disorders

Dweik et al. (15) highlighted a significant relationship between short sleep duration and obesity, noting that poor sleep quality was a predictor of both obesity and depressive symptoms as assessed by the Mood Scale Patient Health Questionnaire (MSPHQ) (15).

### Socio-cultural influences

Several studies emphasized the impact of socio-cultural factors on the relationship between obesity and mental health. For example: Alabdullgader et al. (16) reported that societal pressure and stigmatization related to body size were significant contributors to low self-esteem and mood swings among obese students (16).

## Discussion

This discussion synthesizes findings from a systematic review of nine studies to elucidate the interconnectedness of various variables related to obesity and mental health among university students in Gulf Cooperation Council (GCC) countries and proposes avenues for future research and interventions.

### Differentiating mental health conditions

The relationship between obesity and mental health among university students in Gulf Cooperation Council (GCC) countries is multifaceted, involving a variety of mental health conditions. In our systematic review, we identified several specific mental health issues that are associated with obesity, rather than treating mental health as a single, homogenous entity.

#### Depression

Several studies highlighted a strong association between obesity and depression among university students. For instance, Mellal et al. (10) found a significant link between perceived weight status and depressive symptoms, suggesting that weight-related stigma may exacerbate depressive feelings (10).

#### Anxiety

Anxiety was also frequently associated with obesity. Bener et al. (17) reported that extreme dieting behaviors, which are more common among obese individuals, were linked to heightened anxiety levels (17). This indicates that the pressure to conform to societal beauty standards may contribute to anxiety disorders.

#### Body image dissatisfaction

Body image dissatisfaction emerged as a critical factor in the relationship between obesity and mental health. Studies such as those by Al-Sayegh et al. (13) and Pathath et al. (14)emphasized that negative body image perceptions significantly contribute to psychological distress among obese students (13, 14).

#### Sleep disorders

Short sleep duration and poor sleep quality were found to be predictors of both obesity and mood disorders, including depression and anxiety. Dweik et al. (15) identified a bidirectional relationship where poor sleep exacerbates mental health problems and vice versa (15).

Understanding these specific mental health conditions allows for more targeted interventions. For instance, programs addressing body image issues should include components that promote positive self-esteem and healthy attitudes towards weight. Similarly, interventions aimed at reducing depression and anxiety should consider the underlying causes related to obesity and societal pressures (14).

### Prevalence of obesity and its impact on mental health

Consistent with global trends, the prevalence of overweight and obesity among university students in the GCC countries is alarmingly high (13). The included studies reported a mean prevalence rate of 29.4%. This high prevalence is particularly concerning, as obesity has been consistently linked to various mental health problems, including depression, anxiety, and low self-esteem (10, 16). The findings suggest that addressing obesity among university students is imperative for both physical and psychological well-being.

#### Contributing factors to the relationship between obesity and mental health

##### Dieting behaviors and body image dissatisfaction

The association between extreme dieting behaviors and negative psychological outcomes, such as body image dissatisfaction and psychological distress, has been well-documented in previous research (18). The pressure to conform to societal standards of beauty and thinness may contribute to disordered eating patterns and exacerbate mental health issues among university students in the GCC countries. Interventions targeting body image concerns and promoting healthy attitudes towards food and weight are warranted (19).

##### Sleep patterns

Short sleep duration emerged as a significant predictor of both obesity and mood depression among adolescents in the UAE (15). This finding underscores the bidirectional relationship between sleep and mental health, with poor sleeps quality exacerbating mental health problems and vice versa (15). Given the importance of adequate sleep for overall well-being, interventions aimed at improving sleep hygiene and addressing sleep disorders may have positive implications for mental health outcomes among university students.

##### Socio-cultural influences

The socio-cultural context plays a crucial role in shaping perceptions of body image and influencing health behaviors among university students in the GCC countries (14). Societal norms regarding body size and shape may contribute to the stigmatization of obesity and hinder efforts to promote positive body image and self-esteem. Culturally sensitive interventions that take into account the unique socio-cultural context of the region are needed to address these challenges effectively (14).

##### Psychosocial stressors

Researchers in Saudi Arabia found a significant association between psychosocial stressors like mood swings, social pressure, and low self-esteem and obesity among university students (16). These findings highlight the importance of considering the psychological well-being of students in conjunction with their physical health. Addressing psychosocial stressors through counseling services, peer support programs, and stress management interventions may help mitigate the negative impact of obesity on mental health.

#### Gender differences

Gender differences in the obesity-mental health relationship warrant attention in future research and intervention efforts. Studies have shown variations in body image perception and coping mechanisms between male and female students (20). Understanding these gender-specific differences is crucial for developing tailored interventions that address the unique needs of male and female students effectively.

#### Cultural nuances

Cultural nuances play a pivotal role in shaping health behaviors and perceptions among university students in GCC countries (21). Tailoring interventions to align with cultural values and beliefs can enhance their acceptability and effectiveness (21). Collaborative efforts between researchers, policymakers, and community stakeholders are essential for developing contextually relevant interventions that resonate with the target population.

#### Methodological considerations

While the systematic review provides valuable insights, several methodological considerations merit attention. The heterogeneity of study designs, measurement tools, and sampling techniques across included studies may limit the comparability and generalizability of findings. Future research should adopt standardized methodologies to facilitate robust comparisons and enhance the quality of the evidence.

### Limitations

Despite the comprehensive nature of this systematic review, several limitations must be acknowledged. First, the heterogeneity of the included studies presents a challenge. The studies varied significantly in terms of their design, sample sizes, measurement tools for obesity and mental health outcomes, and the specific populations they examined. This variability may limit the comparability and generalizability of our findings.

Second, the reliance on cross-sectional study designs in the majority of the included studies precludes the ability to draw causal inferences about the relationship between obesity and mental health among university students in the GCC countries. Longitudinal studies are needed to better understand the temporal relationships and potential causal pathways.

Third, there is a potential for publication bias, as our search was restricted to peer-reviewed articles published in English. Studies with null or negative findings may be underrepresented in the literature, potentially skewing our results.

Fourth, the use of self-reported measures for both obesity and mental health outcomes in many of the included studies may introduce bias. Self-reported data are subject to inaccuracies due to social desirability bias, recall bias, and reporting errors. Objective measures and validated assessment tools should be prioritized in future research to enhance the accuracy of findings.

Fifth, the socio-cultural context unique to each GCC country may influence the generalizability of our results. While this review highlights common factors across the GCC region, there are likely country-specific nuances that were not captured. Future studies should consider the specific cultural and environmental factors within each GCC country to provide more tailored insights.

Lastly, while we aimed to include all relevant studies up to February 28, 2024, there is always a possibility that newer studies or studies missed in our search may provide additional insights not covered in this review. Continuous updates to systematic reviews are necessary to incorporate the most current evidence.

Despite these limitations, our review provides valuable insights into the complex relationship between obesity and mental health among university students in the GCC countries, underscoring the need for comprehensive, culturally sensitive interventions and further research efforts.

### Future research directions

Longitudinal studies are warranted to elucidate temporal relationships and causal pathways between obesity and mental health outcomes among university students in GCC countries. Longitudinal designs allow for the assessment of dynamic changes over time, offering insights into the trajectory of obesity-related mental health risks (22). Additionally, interventions targeting modifiable risk factors, such as diet, physical activity, and stress management, hold promise for mitigating the burden of obesity and associated mental health issues (23). Multifaceted interventions incorporating lifestyle modifications, psychoeducation, and behavioral therapies offer holistic approaches to promoting well-being among university students.

## Conclusion

The systematic review on the complex interplay between obesity and mental health among university students in the Gulf Cooperation Council (GCC) countries revealed insightful findings. The included studies, spanning various GCC nations and employing cross-sectional designs, shed light on the prevalence of overweight/obesity, lifestyle practices, psychological outcomes, and socio-cultural influences among university populations. Key findings from the synthesized evidence highlight the significant impact of obesity on mental health outcomes, with factors such as dieting behaviors, body image dissatisfaction, sleep patterns, and socio-cultural stressors playing crucial roles. For instance, studies identified associations between extreme dieting and psychological distress, as well as between short sleep duration, obesity, and depressive symptoms. Additionally, studies implicated socio-cultural changes in the rising obesity rates among university students in Saudi Arabia, underscoring the need for further research and targeted interventions in this area. Overall, the systematic review underscores the importance of adopting comprehensive approaches to address the intertwined nature of obesity and mental health among university students in the GCC countries. These findings have implications for public health policy and practice, highlighting the importance of promoting healthy lifestyle behaviors, addressing psychosocial stressors, and fostering supportive environments to improve the overall well-being of university populations in the region. To mitigate the adverse effects of obesity on mental health and promote resilience among university students in the GCC countries, we need to continue our research efforts and implement evidence-based interventions. By addressing these interconnected challenges, stakeholders can work towards building healthier and more resilient communities, ultimately enhancing the overall quality of life for individuals in the region.

## Data availability statement

The original contributions presented in the study are included in the article/Supplementary material, further inquiries can be directed to the corresponding author.

## Author contributions

AJ: Conceptualization, Data curation, Formal analysis, Funding acquisition, Methodology, Supervision, Visualization, Writing – original draft, Writing – review & editing. SAb: Methodology, Writing – review & editing. SAl: Data curation, Formal analysis, Writing – original draft, Writing – review & editing.

## References

[1] Blaine B. Does depression cause obesity? A meta-analysis of longitudinal studies of depression and weight control. J Health Psychol. (2008) 13:1190–7. doi: 10.1177/1359105308095977, PMID: 18987092

[2] FS L. Overweight, obesity, and depression: a systematic review and meta-analysis of longitudinal studies. Arch Gen Psychiatry. (2010) 67:220–9. doi: 10.1001/archgenpsychiatry.2010.2, PMID: 20194822

[3] Musaiger AO. Overweight and obesity in eastern mediterranean region: prevalence and possible causes. J Obes. (2011) 2011:407237. doi: 10.1155/2011/407237, PMID: 21941635 PMC3175401

[4] Ng M Fleming T Robinson M Thomson B Graetz N Margono C . Global, regional, and national prevalence of overweight and obesity in children and adults during 1980–2013: a systematic analysis for the global burden of disease study 2013. Lancet. (2014) 384:766–81. doi: 10.1016/S0140-6736(14)60460-8, PMID: 24880830 PMC4624264

[5] Al-Rethaiaa AS Fahmy A-EA Al-Shwaiyat NM. Obesity and eating habits among college students in Saudi Arabia: a cross sectional study. Nutr J. (2010) 9:1–10. doi: 10.1186/1475-2891-9-39, PMID: 20849655 PMC2949783

[6] Mokdad AH Jaber S Aziz MIA AlBuhairan F AlGhaithi A AlHamad NM . The state of health in the Arab world, 1990–2010: an analysis of the burden of diseases, injuries, and risk factors. Lancet. (2014) 383:309–20. doi: 10.1016/S0140-6736(13)62189-3, PMID: 24452042

[7] Stallman HM. Psychological distress in university students: a comparison with general population data. Aust Psychol. (2010) 45:249–57. doi: 10.1080/00050067.2010.482109

[8] Wells G Shea B O’Connell D Peterson J Welch V Losos M . Newcastle-Ottawa quality assessment scale. Ottawa Hosp Res Inst. (2014) 3:2–4.

[9] Page MJ McKenzie JE Bossuyt PM Boutron I Hoffmann TC Mulrow CD . The PRISMA 2020 statement: an updated guideline for reporting systematic reviews. Int J Surg. (2021) 88:105906. doi: 10.1136/bmj.n71 33789826

[10] Mellal A Albluwe T Al-Ashkar D. The prevalence of depressive symptoms and its socioeconomic determinants among university students in Al Ain, UAE. Int. J. Pharm. Pharm. Sci. (2014) 6:309–12.

[11] Schulte SJ Thomas J. Relationship between eating pathology, body dissatisfaction and depressive symptoms among male and female adolescents in the United Arab Emirates. Eat Behav. (2013) 14:157–60. doi: 10.1016/j.eatbeh.2013.01.015, PMID: 23557812

[12] Bener A Kamal A Tewfik I Sabuncuoglu O. Prevalence of dieting, overweight, body image satisfaction and associated psychological problems in adolescent boys. Nut Food Sci. (2006) 36:295–304. doi: 10.1108/00346650610703144

[13] Al-Sayegh N Al-Enezi K Nadar M Dean E. Health status, behaviors, and beliefs of health sciences students and staff at Kuwait University: toward maximizing the health of future health professionals and their patients. Int J Environ Res Public Health. (2020) 17:8776. doi: 10.3390/ijerph17238776, PMID: 33255967 PMC7730932

[14] Pathath AW Lone MA Abdulaziz M Al Qurini AA. Obesity and mental health among university students in Saudi Arabia. Int J Indian Psychȯl. (2017) 5. doi: 10.25215/0501.063

[15] Al Dweik R Sheble Y Ramadan H Issa H Sheble A. The association between sleeping behavior, obesity, psychological depression, and eating habits among adolescents in the emirate of Abu Dhabi-United Arab Emirates. PLoS One. (2022) 17:e0269837. doi: 10.1371/journal.pone.0269837, PMID: 36040982 PMC9426872

[16] Alabdullgader AA Alaboudi RS Almatrudi NS Alshammasi M Alsamaani AS Aldhubayb ZK . Psycho-social problems of obesity among male students in Qassim university in Saudi Arabia. Int J Adv Res. (2017) 5:975–9. doi: 10.21474/IJAR01/2905

[17] Bener A Ghuloum S Abou-Saleh MT. Prevalence, symptom patterns and comorbidity of anxiety and depressive disorders in primary care in Qatar. Soc Psychiatry Psychiatr Epidemiol. (2012) 47:439–46. doi: 10.1007/s00127-011-0349-9, PMID: 21293844

[18] Bener A Tewfik I. Prevalence of overweight, obesity, and associated psychological problems in Qatari’s female population. Obes Rev. (2006) 7:139–45. doi: 10.1111/j.1467-789X.2006.00209.x, PMID: 16629870

[19] Alleva JM Sheeran P Webb TL Martijn C Miles E. A meta-analytic review of stand-alone interventions to improve body image. PLoS One. (2015) 10:e0139177. doi: 10.1371/journal.pone.0139177, PMID: 26418470 PMC4587797

[20] Rababa M Al-Sabbah S. Nurses’ pain assessment practices for cognitively intact and impaired older adults in intensive care units. Dementia and geriatric cognitive disorders extra. (2022) 12:115–21. doi: 10.1159/000525477 35950149 PMC9294931

[21] Al-Hazzaa HM Abahussain NA Al-Sobayel HI Qahwaji DM Musaiger AO. Lifestyle factors associated with overweight and obesity among Saudi adolescents. BMC Public Health. (2012) 12:1–11. doi: 10.1186/1471-2458-12-354 22591544 PMC3433359

[22] Shabana HA Khafaga Tamer Al-Hassan Hamdan Alqahtani Shaykah. Medicinal plants diversity at king Salman bin Abdulaziz Royal Natural Reserve in Saudi Arabia and their conservation management. J. Med. Plants Res. (2023) 17:292–304. doi: 10.5897/JMPR2023.7317

[23] AlBuhairan FS Tamim H Al Dubayee M AlDhukair S Al Shehri S Tamimi W . Time for an adolescent health surveillance system in Saudi Arabia: findings from “Jeeluna”. J Adolesc Health. (2015) 57:263–9. doi: 10.1016/j.jadohealth.2015.06.009, PMID: 26299553

[24] Khadri FA Gopinath VK Hector MP Davenport ES. Impact of demographic factors, obesity, and oral health status on self-esteem among school-going children in United Arab Emirates: a cross-sectional study. J Int Soc Prevent Commun Dent. (2020) 10:329–35. doi: 10.4103/jispcd.JISPCD_422_19, PMID: 32802780 PMC7402253

